# NOD2 Genotypes Affect the Symptoms and Mortality in the Porcine Circovirus 2-Spreading Pig Population

**DOI:** 10.3390/genes12091424

**Published:** 2021-09-16

**Authors:** Kasumi Suzuki, Hiroki Shinkai, Gou Yoshioka, Toshimi Matsumoto, Junji Tanaka, Noboru Hayashi, Haruki Kitazawa, Hirohide Uenishi

**Affiliations:** 1 Swine and Poultry Research Department, Gifu Prefectural Livestock Research Institute, Seki 501-3924, Japan; suzuki-kasumi@pref.gifu.lg.jp (K.S.); yoshioka-go@pref.gifu.lg.jp (G.Y.); tanaka-junji@pref.gifu.lg.jp (J.T.); hayashi-noboru@pref.gifu.lg.jp (N.H.); 2 Food and Feed Immunology Group, Laboratory of Animal Food Function, Graduate School of Agricultural Science, Tohoku University, Sendai 980-8572, Japan; 3 Livestock Immunology Unit, International Education and Research Center for Food Agricultural Immunology (CFAI), Graduate School of Agricultural Science, Tohoku University, Sendai 980-8572, Japan; 4 National Institute of Animal Health, National Agriculture and Food Research Organization (NARO), Tsukuba 305-0856, Japan; sinkai@affrc.go.jp; 5 Institute of Agrobiological Sciences, National Agriculture and Food Research Organization (NARO), Tsukuba 305-8634, Japan; mtoshimi@affrc.go.jp

**Keywords:** disease resistance, pattern recognition receptors, post-weaning multisystemic wasting syndrome, PCV2, swine

## Abstract

The nucleotide oligomerization domain (NOD)-like receptor 2 (NOD2) is an intracellular pattern recognition receptor that detects components of peptidoglycans from bacterial cell walls. NOD2 regulates bowel microorganisms, provides resistance against infections such as diarrhea, and reduces the risk of inflammatory bowel diseases in humans and mice. We previously demonstrated that a specific porcine *NOD2* polymorphism (*NOD2*-2197A > C) augments the recognition of peptidoglycan components. In this study, the relationships between porcine *NOD2*-2197A/C genotypes affecting molecular functions and symptoms in a porcine circovirus 2b (PCV2b)-spreading Duroc pig population were investigated. The *NOD2* allele (*NOD2*-2197A) with reduced recognition of the peptidoglycan components augmented the mortality of pigs at the growing stage in the PCV2b-spreading population. Comparison of *NOD2* allele frequencies in the piglets before and after invasion of PCV2b indicated that the ratio of *NOD2*-2197A decreased in the population after the PCV2b epidemic. This data indicated that functional differences caused by *NOD2*-2197 polymorphisms have a marked impact on pig health and livestock productivity. We suggest that *NOD2*-2197CC is a PCV2 disease resistant polymorphism, which is useful for selective breeding by reducing mortality and increasing productivity.

## 1. Introduction

The innate immune system is armed with many types of pattern recognition receptors (PRRs) that recognize pathogen-associated molecular patterns (PAMPs). PRRs detect the invasion of pathogens such as bacteria and viruses at the very early stage of infection by initiating a comprehensive immune response including T and B cell activity in acquired immunity through the induction of proinflammatory cytokines and interferons. PRRs play their roles in various cellular locations, such as the cell surface and intracellular organelles. Some examples of PRRs include Toll-like receptors (TLRs), retinoic acid-inducible gene I (RIG-I)-like helicases (RLHs), C-type lectin-like receptors (CLRs), and nucleotide oligomerization domain (NOD)-like receptors [1]. Polymorphisms in PRRs frequently affect resistance to infectious diseases and the onset and exacerbation of autoimmune diseases in humans [2].

We have demonstrated that PRR genes have many polymorphisms that affect coding amino acids in various pig populations [3,4,5,6]. Some of these polymorphisms affect molecular functions such as PAMP recognition [7]. For instance, a *TLR5* (1205C > T) polymorphism attenuates the response of TLR5 to recognize the flagellin of *Salmonella* Choleraesuis [8]. Similarly, the *TLR5*-1205T genotype has a reduced ability to detect the pathogen *Salmonella* Typhimurium in pigs, resulting in severe diarrhea in comparison with normal TLR5 (*TLR5*-1205C) indicating that this polymorphism may be used to improve disease resistance [9].

Nucleotide oligomerization domain (NOD)-like receptors (NLRs) are cytosolic PRRs that consist of an NOD, leucine-rich repeats, and a signal transduction domain. NOD1 and NOD2 are members of the NLR family that recognize key structural components of the peptidoglycan of bacterial cell walls. NOD1 recognizes γ-D-glutamyl-meso-diaminopimelic acid (iE-DAP), which is mainly observed in gram-negative bacteria and some gram-positive bacteria [10]. NOD2 recognizes muramyl dipeptide, which is a common motif in the peptidoglycan of gram-negative and gram-positive bacteria [11].

Viral infection augments signaling through NOD1/NOD2 pathways to oppose secondary bacterial infection [12]. Furthermore, NOD2 is involved in the activation of type I interferon by the detection of single-stranded RNA derived from viruses [13,14]. *NOD1* and *NOD2* have polymorphisms that affect ligand recognition in pig populations. *NOD1* mutations (1922G > A and 2752G > A) abrogate the recognition of the iE-DAP structure [15], while a 2197 (A > C) mutation in *NOD2* promotes the recognition of muramyl dipeptide [16]. The *NOD1*/*NOD2* polymorphisms affecting ligand recognition are broadly distributed in commercial pig populations, and are attracting interest in pig breeding strategies aimed at disease resistance.

The prevalence of postweaning multisystemic wasting syndrome (PMWS) has been observed worldwide since its first detection in Canada [17], and porcine circovirus type 2 (PCV2) was recognized as the causal agent of PMWS [18,19]. PCR screening detected PCV2 DNA in more than 80% of pigs in farms throughout Japan [20]. Although PCV2 infection in pigs is rarely fatal, secondary infections increase the severity of the symptoms caused by PCV2-induced immunosuppression (PCV2-associated diseases; PCVAD) [21]. PCV2 infection frequently causes decreased pig growth rate and increased pig mortality at weaning and early fatting stages resulting in huge economic losses in the livestock industry [22].

In this study, the relationship between *NOD2* genotypes and mortality in a Duroc pig population infected with PCV2 was investigated.

## 2. Materials and Methods

### 2.1. Ethics Statement, Pig Population and Sampling

All handling of pigs and pig transport was approved by the Animal Care and Use Committee of the Gifu Prefectural Livestock Research Institute (No. R03-107).

A *Sus scrofa domesticus* Duroc breed population named Iris Nagara was established by the Gifu Prefectural Livestock Research Institute (GPLRI) and the Aichi Prefectural Livestock Research Center. This study was conducted using a subpopulation of Iris Nagara maintained in the facilities of GPLRI from 2008 to 2013. The population used in this study was reared separated from other pigs throughout the experiment and the genetic source thus did not change. We did not select pigs based on genotypes or disease resistance in the population. The pigs used throughout this study were maintained in the identical environment and location, and were porcine reproductive and respiratory syndrome virus (PRRSV)-free throughout the study. The mortality and body weight at day 60 after birth of each pig was recorded. The mortality rate was calculated based on the number of deaths 60 days after birth. Vaccination of PCV2 with Circovac^®^ (Ceva, Libourne, France) or CircoFLEX^®^ (Boehringer Ingelheim, Ingelheim, Germany) was conducted in 151 animals born in 2012 and in those born in 2013. The remaining 949 animals were not vaccinated. Tissue and blood samples were collected at slaughter or during the rearing management procedure. Genomic DNA was extracted from the tissues and blood using an Easy DNA™ gDNA purification kit (Invitrogen, Carlsbad, CA, USA). Viral DNA was extracted from blood samples using a NucleoSpin^®^ virus mini kit (Macherey-Nagel, Düren, Germany).

### 2.2. Comparison of Capsid Gene Sequence of PCV2

Complete sequences of the PCV2 capsid genes were amplified from genomic DNA extracted from blood using forward (5′-CCATGCCCTGAATTTCCATA-3′), and reverse (5ʹ-GGGCACCAAAATACCACTTC-3′) PCR primers [23] using an EmeraldAmp PCR master mix (Takara Bio, Otsu, Japan). PCR was initiated by denaturation for 3 min at 94 °C, followed by 35 cycles of 30 s at 95 °C, 30 s at 60 °C, and 90 s at 72 °C. The PCR cycles were followed by an additional extension of 10 min at 72 °C. Amplified PCR products were sequenced using an Applied Biosystems 3730xl DNA analyzer with the BigDye Terminator 3.1 cycle sequencing kit (Thermo Fisher Scientific, Palo Alto, CA, USA). Phylogenetic analysis was performed by the neighbor-joining method [24] using the program ClustalX2 [25].

### 2.3. Genotypes of the NOD2 Gene

DNA fragments surrounding the *NOD2* polymorphism (2197A/C) affecting molecular function were amplified using genomic DNA of the pigs with primers (forward: 5′-GGTGTCTGAGAAGGCTCTGC-3′; reverse: 5′-TTGCAGACGTTGAGACAAGG-3′) using AmpliTaq Gold DNA polymerase (Thermo Fisher Scientific). PCR was initiated by denaturation for 10 min at 94 °C, followed by 45 cycles of 30 s at 95 °C, 30 s at 55 °C, and 1 min at 72 °C. The PCR cycles were followed by an additional extension of 5 min at 72 °C. The obtained fragments were sequenced as described above. The genotypes were confirmed using sequencing reads by automated single nucleotide polymorphism (SNP) detection with PolyPhred [26], and manual inspection using Consed [27] after assembly by Phred basecaller and Phrap assembler [28,29].

### 2.4. Computational Analysis

Significant differences in mortality rates were examined using the Chi-square test. Survival rates were determined using the log-rank test. Statistical analyses were performed using R (https://www.r-project.org/, accessed on 30 July 2021)

## 3. Results

### 3.1. Phylogenetic Differences in PCV2 Strains before and after Wasting Symptoms and Frequent Mortality

**Figure 1:**
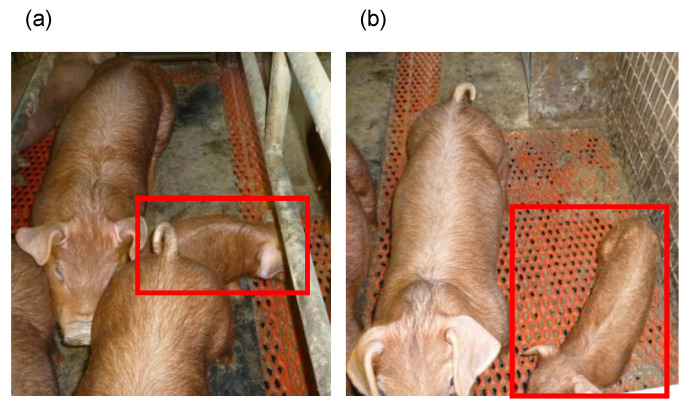
Representative symptoms of PCVAD observed in the pig population. Photographs (**a**) and (**b**) show littermates. Pigs indicated by red rectangles demonstrated typical growth retardation in comparison with other littermates.

Many disorders were observed in the populations maintained in the pig facilities of the GPLRI from 2009 including wastage, diarrhea, and frequent death (Figure 1). The mortality rate of the pig population increased from 6.6% in 2008 to 33.6% in 2010. The severe epidemic settled down in 2011; however, the mortality rate did not restore the level comparable with that before the epidemic. After PCV2 vaccination, the mortality rate dropped to 5.8% in 2013 (Table 1). Male pigs showed a slightly higher mortality than female pigs, although relationships of the high mortality with PCVAD were unclear. We also evaluated the effects of the epidemic by growth retardation at day 60 after birth. The number of pigs with growth retardation increased after 2009, and more than 20% of pigs were regarded as “boney” (less than 75% of the average weight) in 2010 and 2011 (Tables S2 and S3).

**Figure 2:**
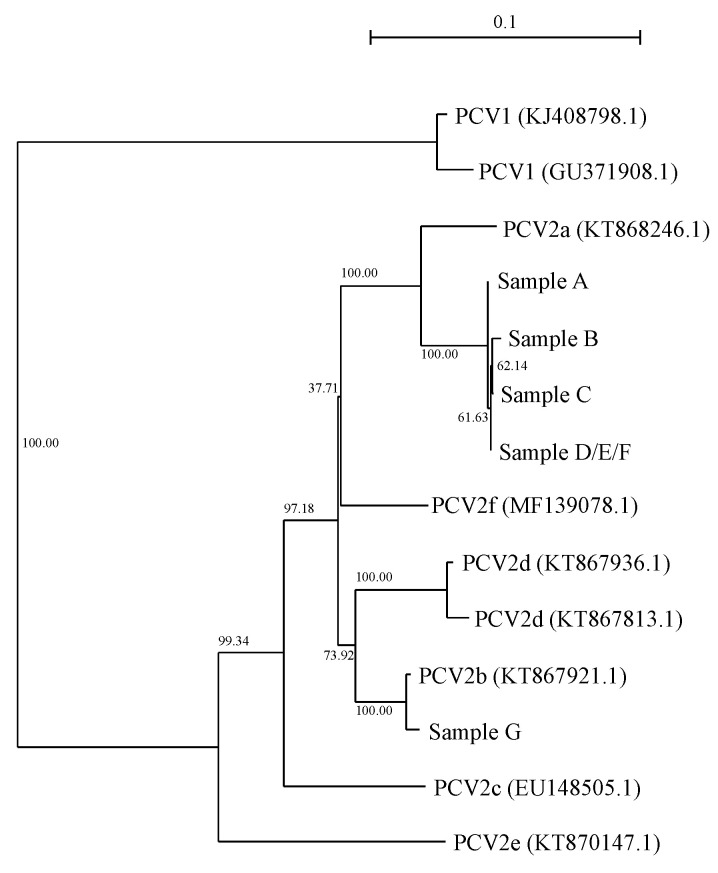
Phylogenetic tree of complete coding sequences of *PCV2* capsid gene detected in the pig population (sample A–G) and representative sequences derived from different genotypes of PCV2 [35,36]. Birth dates of the samples and accession numbers in DDBJ/EMBL/GenBank nucleotide databases of the capsid gene sequences are as follows: A, 3 April 2007/LC637748.1; B, 29 October 2008/LC637749.1; C, D, E and F, 18 February 2009/LC637750.1, LC637751.1, LC637752.1, LC637753.1; G, 13 April 2012/LC637754.1. Bootstrap values for 10,000 replicates are shown as percentages beside the branches. The scale of the branch length is shown at the top of the figure.

PCV2a infection was detected in the pig population before the emergence of wasting symptoms, while only PCV2b was detected after wasting symptoms were observed. The detected capsid gene sequence had a high similarity to PCV2b, the virulence of which was found to be higher than that of PCV2a in a previous report [30]. Although the genotypes determined by the capsid gene do not necessarily define the virulence of PCV2 [31], genotypes switching seemed to synchronize with PCVAD emergence. We could not obtain samples suitable for the extraction of viral DNA in 2010; therefore, we could not isolate PCV2 capsid gene sequences and determine the genotype of PCV2 in samples from that year. However, the typical symptoms of PCVAD, such as growth retardation (Figure 1) and an increased pig mortality at weaning and early fatting stages, which were consecutively observed after 2009, were not observed during the period when PCV2a was detected. This suggests that PCV2b invasion was the causative event for the increased mortality rate. This strongly implied that the wasting symptoms and frequent death were due to PCVAD, caused by the invasion of the virulent PCV2b strain into the population (Figure 2).

### 3.2. Marked Differences in NOD2 Genotype Frequency before and after the Emergence of PCVAD

The pig population had a nonsynonymous polymorphism at position 2197 (A/C; Ser/Arg at position 733 in the deduced amino acid sequence) in the coding sequence of *NOD2*. In Western breeds, the majority of pigs have the *NOD2*-2197A allele [3] (GenBank accession number: AB426547.1), which shows weakened molecular function to recognize muramyl dipeptide. The Duroc breed frequently has the *NOD2*-2197C allele, which augments the recognition of ligands [16]. The reference genome sequence of *Sus scrofa* was constructed with a female Duroc pig [32] and had the *NOD2*-2197C allele, in accordance with the high frequency of *NOD2*-2197C in Duroc pigs [3,16].

Previous reports show that castrated male pigs were more susceptible to PCVAD than female pigs [33,34], suggesting that a sex-effect may skew the results related to the association between SNP and PCVAD susceptibility. Furthermore, we could prepare more samples of female pigs as compared to male pigs because of a preservation of samples. Therefore, we used mainly female pigs in this study. More than 80 female individuals were randomly selected from each group born in different years and genotyped for the *NOD2*-2197 SNP. In pigs born before and during the emergence of PCVAD symptoms, the frequency of *NOD2*-2197A was approximately 40%. After PCVAD emergence (2012), the *NOD2*-2197A frequency significantly decreased to 23% (*p* < 0.01) in all alleles (Table 2). Differences in mortality due to PCVAD among pigs with different *NOD2* genotypes might reflect allele frequencies of the *NOD2* gene of progenies. Any selection based on genetic information or disease-resistance indices was not conducted in the pig population of this study; therefore, the decrease in *NOD2*-2197A frequency might be caused by a high mortality of parent pigs with *NOD2*-2197A in 2009 to 2011. Similar results were obtained from the comparatively smaller number of male individuals (Table S4).

### 3.3. Significant Influence of NOD2 Genotypes on Pig Mortality during the Spread of PCV2b

**Figure 3:**
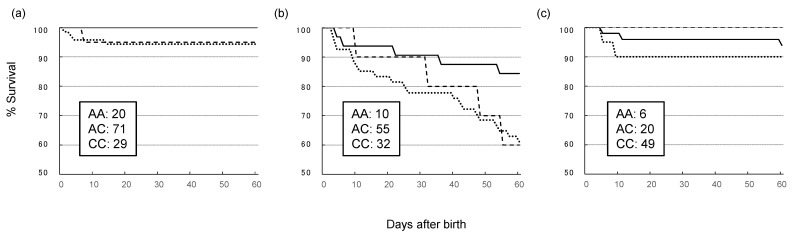
Survival curves of pigs before the invasion of PCV2b in 2008 (**a**), after invasion in 2010 (**b**), and after vaccination against PCV2 in 2012 (**c**). Survival rates of pig populations with AA, AC, and CC are indicated by dashed, dotted, and solid lines, respectively. The legend indicates the numbers of animals investigated with the respective genotypes.

The mortality rates of pigs before PCV2b invasion were comparable between the *NOD2* genotypes (Figure 3a). After PCV2b invasion, approximately 40% of the pigs with *NOD2*-2197AA and *NOD2*-2197AC genotypes died within 60 days after birth in 2010. In contrast, 84% of the pigs with *NOD2*-2197CC genotypes survived during the same observation period (Figure 3b) (log-rank test, *p* = 0.02). Significant differences between the mortalities of pigs with *NOD2*-2197AA and *NOD2*-2197AC were not observed. Meanwhile, no significant difference in survival rates among pigs with different genotypes was observed following vaccination against PCV2 (Figure 3c). We also investigated the effect of *NOD2* genotypes on growth retardation at day 60 after birth. Before the PCVAD epidemic in 2008, a marked difference was not observed among pigs with different *NOD2* genotypes. Due to the limitation of samples because of the death of pigs due to PCVAD, significant differences between *NOD2* genotypes were not observed; however, the percentage of pigs with growth retardation was markedly reduced by the *NOD2*-2197C allele (Figure S1).

## 4. Discussion

In this study, we demonstrated that SNP in *NOD2* markedly affected susceptibility to PCVAD in a pig population. In the investigated population, the protective effect by vaccination against PCVAD was more prominent than that by genetic selection of the SNP in the *NOD2* gene. However, the results in this study indicate the possibility to improve the resistance against infectious diseases by selecting a specific allele of immune genes in pigs. Furthermore, *NOD2* is involved in the immune response to both bacterial and viral infections, suggesting the possibility to improve the resistance to other diseases by selection of *NOD2* alleles. We demonstrated a definite effect of *NOD2*-2197 polymorphisms on the recognition of bacterial components in a previous in vitro study [16]; however, the effect of the polymorphism on viral immunity has not been elucidated. Clarifying the molecular fundamentals of the protection effect against PCVAD related to *NOD2* genotypes will shed light on *NOD2* functioning in the viral immunity in pigs.

An *NOD2* 2197 polymorphism affecting ligand recognition is associated with mortality in the pig population. Infection with virulent PCV2 causes immune suppression in the host animal, which may result in secondary infection with bacteria and/or viruses into the pig population [37,38]. For example, PCV2b infection resulted in pigs becoming susceptible to secondary infection with *Lawsonia intracellularis* with many pigs showing emaciation, which may be caused by porcine proliferative enteritis [39].

NOD2 plays a key role in the response against bacteria by recognizing the components of bacterial cell wall peptidoglycan, with NOD2 defects causing the onset or severity of bacterial infections in experimental animal models [40,41,42]. The porcine *NOD2* polymorphism (2197A/C) affects recognition of the ligand [3,16]. Therefore, the diminished response caused by the *NOD2* mutation may result in the failure of protection against secondary infection, despite viral infection augmenting the expression of *NOD2* and its signaling receptor *RIP2* (receptor-interacting-serine/threonine-protein kinase 2) [12].

We must consider another possibility regarding the role of the *NOD2* polymorphism in PCV2b infection. Several studies have indicated that NOD2 participates in the cellular immune response to viruses [13,14]. Therefore, the impaired function of NOD2 may cause immunosuppression through negatively affecting the response to PCV2b infection resulting in an insufficient response to secondary infection. Under experimental conditions, sole PCV2 infection induces diarrhea symptoms [43]; therefore, the dynamics of pathogens in the farms should be investigated in individual cases.

NOD2 recognizes bacterial peptidoglycans and induces an inflammatory response [44]. NOD2 is involved in the digestion of bacteria invading cells by autophagy processes [45,46]. Furthermore, NOD2 contributes to a decreased risk of developing Crohn’s disease by regulating excessive signal transduction by TLRs [47] and production of interleukin 10 [48,49]. Meanwhile, a loss-of-function mutation in *NOD2* is a widely accepted risk factor for Crohn’s disease in humans [50,51,52]. The *NOD2*-2197C genotype mildly augments the molecular function of NOD2 in comparison with *NOD2*-2197A [16], which may contribute to a moderate immune response to exert anti-microbial activity of the hosts and avoid excessive inflammation, which exacerbates pneumonia [53]. The *NOD2*-2197C genotype is broadly distributed among different representative pig breeds, including Landrace, Large White, and Duroc [3]. Furthermore, *NOD2*-2197C is the predominant genotype in Japanese wild boars [3,16]. We previously showed that *NOD2*-2197C improves ligand recognition compared with other NOD2 polymorphisms in Duroc pigs commonly used in Japanese farming [16]. This suggests that the *NOD2*-2197C genotype does not confer a harmful effect on pig health or other economic traits, although further investigations are required.

In this study, *NOD2*-2197CC had a marked effect in decreasing the mortality caused by PCVAD. On the other hand, a reduction in the mortality rate of pigs with a heterozygous genotype (*NOD2*-22197AC) in comparison with *NOD2*-2197AA was not indicated in this population. The Duroc breed, which was used in this study, is mainly used for production of meat pigs by mating with crossbred white pigs; therefore, if *NOD2-*2197AC meat pigs show a higher disease resistance than *NOD2*-2197AA pigs, the selection of Duroc pigs with *NOD2*-2197CC will contribute to promote the production efficiency of pork meat. On the other hand, a positive effect on growth retardation by a single *NOD2*-2197C allele can be expected based on the data in this study, although the data was limited. Further investigation on disease resistance by a single *NOD2*-2197C allele should be conducted.

It is noteworthy that a difference in mortality between male and female pigs was observed in the population in this study. The mortality rate at the peak of the epidemic (2010) was not markedly different between males and females; however, in the beginning (2009) and settled-down (2011) stages of the epidemic, male pigs had a higher mortality than female pigs. Interestingly, in the settled-down stage of the epidemic (2011), pigs with growth retardation were more frequently males than females. Males might be more severely be infected than females. The number of male pigs with growth retardation was markedly smaller than that of females in 2010, when the epidemic approached the peak, possibly because the infected male pigs were dead at the early rearing stage. Differences in the effects of SNPs on disease resistance between sexes should be further investigated.

There is another *NOD* gene that has polymorphisms affecting its molecular function. *NOD1* has two polymorphisms, *NOD1*-1922G/A and *NOD1*-2752G/A. In both of the polymorphisms, the A alleles show marked decrease of ligand recognition ability [15]. However, the two SNPs in *NOD1* were not linked to each other; furthermore, a pilot study did not demonstrate an association of *NOD1* SNPs with mortality rates (data not shown). Therefore, we focused on *NOD2* in this study. Further analyses on the association between *NOD1* polymorphisms and the resistance to infectious diseases, particularly bacterial infections, are expected to elucidate the possible contribution of *NOD1* polymorphisms to disease susceptibility.

This study demonstrated the usefulness of the *NOD2*-2197 genotype as a DNA marker for improving disease resistance. Other DNA markers affect pig susceptibility to diarrhea by *Salmonella* (*TLR5*-1205) [8,9], while antibody production after vaccination with *NLRP3*-2906 promotes disease resistance pig breeding [54,55]. Genetic selection of the *NOD2*-2197C genotype may improve resistance to bacterial and/or viral infection, including PCV2b, contributing to the prevention of growth retardation or death during the rearing period due to an enhanced response to the ligand. This is expected to result in an increase in the productivity and quality of pork products by improving the health of the pig population.

## 5. Conclusions

In this study, we demonstrated that pigs with *NOD2*-2197CC are more resistant to PCVAD. A promising candidate DNA marker (*NOD2*-2197C) is presented for improving disease resistance and, particularly, protection against PCVAD in pig breeding. We anticipate this will result in a reduction in the hygienic costs and an improved productivity and quality of the pork. The effect of heterozygous *NOD2*-2197AC pigs on disease resistance should be evaluated, and further investigations are expected to show the efficacy of the DNA marker on other infectious diseases.

## Figures and Tables

**Table 1:** Mortality of rearing pigs in the Duroc population.

Birth Year	Male			Female			Total		
	Total	Dead	Dead/ Total (%)	Total	Dead	Dead/ Total (%)	Total	Dead	Dead/Total (%)
2008	128	10	7.8	130	7	5.4	258	17	6.6
2009	136	30	22.1	129	23	17.8	265	53	20.0
2010	116	39	33.6	101	34	33.7	217	73	33.6
2011	86	10	11.6	78	7	9.0	164	17	10.4
2012	96 (71)	12 (5)	12.5 (7.0)	100 (80)	6 (5)	6.0 (6.3)	196 (151)	18 (10)	9.2 (6.6)
2013	142 (142)	8 (8)	5.6 (5.6)	153 (153)	9 (9)	5.9 (5.9)	295 (295)	17 (17)	5.8 (5.8)

Numbers of animals inoculated with the PCV2 vaccine are indicated in parentheses. Numbers limited to those with genotyping data for *NOD2*-2197 are indicated in Table S1.

**Table 2:** Allele frequency of *NOD2*-2197 in the Duroc population.

Birth Year	AA	AC	CC	Frequency of a Allele (%)
2008	20	75	29	46.4
2009	23	65	44	42.9
2010	10	58	31	39.4
2011	22	36 *	23	49.4
2012	8	30 **	62 ^††^	23.0 ^‡‡^

Randomly chosen female pigs born between 2008 and 2012 were genotyped. The numbers of animals with the respective genotypes are shown. The frequency of *NOD2*-2197A in all chromosomes of each group is also indicated. Significant decreases (*, *p* < 0.05; **, *p* < 0.01) and increases (^††^, *p* < 0.01) of the alleles compared to those in 2008 are highlighted. A significant change in the frequency of the *NOD2*-2197A allele compared with that in 2008 is shown by a double dagger (^‡‡^, *p* < 0.01).

## Data Availability

Sequence data were deposited in the DDBJ/EMBL/GenBank nucleotide database (accession numbers: LC637748-LC637754).

## Supplementary Materials

The following are available online at https://www.mdpi.com/article/10.3390/genes12091424/s1, Table S1: Mortality of rearing pigs with *NOD2*-2197 genotyping data in the Duroc population, Table S2: Pigs with growth retardation (“boney pigs”) in the Duroc population, Table S3: Pigs with growth retardation (“boney pigs”) that have NOD2-2197 genotyping data in the Duroc population, Table S4: Allele frequency of *NOD2*-2197 in male pigs, Figure S1: Effect of NOD2-2197 genotypes on percentage of pigs with growth retardation.
