# Supplementary material for: NOD2 Genotypes Affect the Symptoms and Mortality in the Porcine Circovirus 2-Spreading Pig Population
Source: Genes (Basel). 2021 Sep 16;12(9):1424. doi: 10.3390/genes12091424 (PMC8469532; doi:10.3390/genes12091424)
Supplement: Supplementary file 1 [file genes-12-01424-s001.zip › genes-1341376-suppl.pdf]

Table S1 Mortality of rearing pigs with *NOD2*-2197 genotyping data in the Duroc population.

Birth year	Male			Female			Total		
	Total	Dead	Dead / total (%)	Total	Dead	Dead / total (%)	Total	Dead	Dead / total (%)
2008	40	4	10.0	120	5	4.2	160	9	5.6
2009	50	10	20.0	129	23	17.8	179	33	18.4
2010	50	21	42.0	97	31	32.0	147	52	35.4
2011	18	8	44.4	78	7	8.9	96	15	15.6
2012	18<br>(14)	5<br>(3)	27.8<br>(21.4)	96<br>(76)	6<br>(5)	6.3<br>(6.6)	114<br>(90)	11<br>(8)	9.6<br>(8.9)
2013	10<br>(10)	1<br>(1)	10.0<br>(10.0)	2<br>(2)	0<br>(0)	0.0<br>(0.0)	12<br>(12)	1<br>(1)	8.3<br>(8.3)

Numbers of animals inoculated with the PCV2 vaccine are indicated in parentheses.

Table S2 Pigs with growth retardation (“boney pigs”) in the Duroc population.

Birth year	Male			Female			Total		
	Total	Boney	Boney / total (%)	Total	Boney	Boney / total (%)	Total	Boney	Boney / total (%)
2008	115	7	6.1	121	8	6.6	236	15	6.4
2009	105	14	13.3	103	12	11.7	208	26	12.5
2010	76	12	15.8	67	21	31.3	143	33	23.1
2011	76	27	35.5	70	14	20.0	146	41	28.1
2012	84<br>(66)	4<br>(3)	4.8<br>(4.5)	94<br>(75)	5<br>(3)	5.3<br>(4.0)	178<br>(141)	9<br>(6)	5.1<br>(4.3)
2013	134<br>(134)	12<br>(12)	9.0<br>(9.0)	144<br>(144)	21<br>(21)	14.6<br>(14.6)	278<br>(278)	33<br>(33)	11.9<br>(11.9)

“Boney” pigs were defined as pigs with less than 75% (16.9 kg) of the average weight of all of pigs at day 60 after birth (22.6 kg). Dead pigs and pigs without weight data were eliminated at day 60 are eliminated. Numbers of animals inoculated with the PCV2 vaccine are indicated in parentheses.

Table S3 Pigs with growth retardation ("boney pigs") that have *NOD2*-2197 genotyping data in the Duroc population.

Birth year	Male			Female			Total		
	Total	Boney	Boney / total (%)	Total	Boney	Boney / total (%)	Total	Boney	Boney / total (%)
2008	36	3	8.3	113	8	7.1	149	11	7.4
2009	38	7	18.4	103	12	11.7	141	19	13.5
2010	29	2	6.9	66	21	31.8	95	23	24.2
2011	10	1	10.0	70	14	20.0	80	15	18.8
2012	13<br>(11)	0<br>(0)	0.0<br>(0.0)	90<br>(71)	5<br>(3)	5.6<br>(4.2)	103<br>(82)	5<br>(3)	4.9<br>(3.7)
2013	9<br>(9)	2<br>(2)	22.2<br>(22.2)	2<br>(2)	0<br>(0)	0.0<br>(0.0)	11<br>(11)	2<br>(2)	18.2<br>(18.2)

"Boney" pigs are defined as pigs with less than 75% (16.9 kg) of the average weight of all of pigs at day 60 after birth (22.6 kg). Dead pigs and pigs without weight data at day 60 after birth are eliminated. Numbers of animals inoculated with the PCV2 vaccine are indicated in parentheses.

Table S4 Allele frequency of *NOD2*-2197 in male pigs.

Birth year	AA	AC	CC	Frequency of A allele (%)
2008	6	21	14	40.2
2009	20††	25	6**	63.7‡‡
2010	5	24	21	34.0
2011	2	11	6	39.4
2012	2	6	13	23.8

The numbers of animals with the respective genotypes are shown and the frequency of *NOD2*-2197A in all chromosomes from each group is also indicated. Significant decreases (**; $P < 0.01$) and increases (††, $P < 0.01$) of alleles in comparison with those in 2008 are highlighted. A significant change in the frequency of the *NOD2*-2197A allele compared with that in 2008 is shown with a double dagger (‡‡; $P < 0.01$).

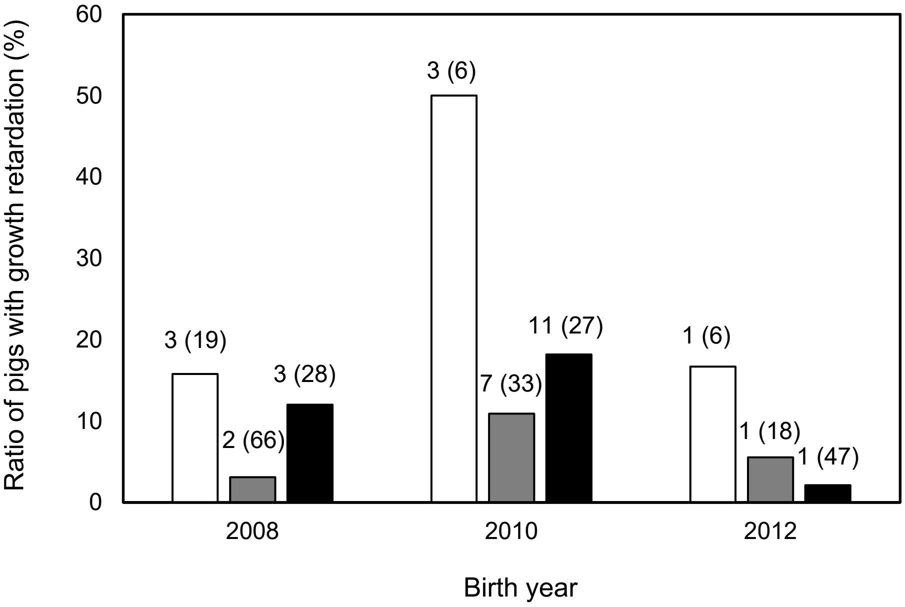

Figure S1 Effect of *NOD2*-2197 genotypes on ratios of pig with growth retardation. Ratios of pigs with weight less than 75 % of the average throughout this study (2008-2013) at day 60 after birth (22.6 kg) in 2008 (before the PCVAD epidemic), 2010 (under the epidemic) and 2012 (after the epidemic) are indicated for the respective *NOD2*-2197 genotypes (white, *NOD2*-2197AA; gray, *NOD2*-2197AC; black, *NOD2*-2197CC). Numbers of “boney” pigs are indicated with total pig numbers (in parentheses). The pigs in 2012 are limited to those with vaccination.
